# HSV-Induced Systemic Inflammation as an Animal Model for Behçet’s Disease and Therapeutic Applications

**DOI:** 10.3390/v10090511

**Published:** 2018-09-19

**Authors:** S. M. Shamsul Islam, Seonghyang Sohn

**Affiliations:** 1 Department of Biomedical Science, Ajou University School of Medicine, Suwon 16499, Korea; shamsulislam21@gmail.com; 2 Department of Microbiology, Ajou University School of Medicine, Suwon 16499, Korea

**Keywords:** Behçet’s disease, herpes simplex virus, animal model, application

## Abstract

Behçet’s disease (BD) affects multiple organs. It is mainly characterized by recurrent oral, skin, and genital aphthous ulcers, and eye involvement. Successful management of BD is increasing, although its etiology remains unclear. A number of etiologies have been proposed, including environmental, genetic, viral, and immunological factors. To understand its complex etiology and improve its management, animal models of BD have been used to enable more effective therapeutic applications with increased clinical significance. An herpes simplex virus (HSV) type 1-induced BD mouse model has shown disease characteristics similar to those seen in BD patients. An HSV-induced BD animal model has been used to test various therapeutic modalities. The applied modalities are several materials that are derived from natural products, conventional therapeutics, and possible biologics. In this review, we provided how they regulate inflammation in an HSV-induced BD model.

## 1. Introduction

Behçet’s disease (BD) is a chronic, relapsing, multi-systemic, and vascular inflammatory disorder that affects many organ systems, including mucocutaneous, ocular, vascular, arthritic, gastrointestinal, and central nervous systems [1]. Until now, the pathogenesis is not conclusive. Several factors, including environmental pollutants, infections, genetic polymorphism, and immune dysregulation, have been suggested as factors affecting the pathogenesis of BD. It seems likely all these factors may contribute together [2]. Herpes simplex virus (HSV) infection [3,4,5,6] is believed to be a triggering factor of BD. HSV-induced BD animal models have been shown to have similar inflammatory responses compared with BD patients, such as mucocutaneous, ocular, vascular, arthritic, and gastrointestinal involvement [7]. Recent progress in the treatment of BD is getting better, but more needs to be done. Using HSV-induced inflammatory BD animal models, therapeutic effects of natural products, and new biological agents have been applied in recent years. They are reviewed and discussed here.

## 2. Etiopathogenesis of Behçet’s Disease

### 2.1. Clinical Significance of HSV

In 1937, Hulusi Behçet proposed that the syndrome might be caused by viral infection, in his first description of BD. However, he could not demonstrate that the virus was HSV in his publication. In 1953, Sezer et al. [5] isolated the virus from ocular fluid of patients. 211-bp HSV-1 DNA fragments and serum anti-HSV-1 antibodies were found in patients with BD [6]. Later, Lee et al. [8] detected HSV DNA in the saliva of patients with BD, and hypothesized that HSV infection might be the trigger of BD. Tojo et al. also detected HSV genome in the skin lesions of BD patients [9], and Eglin et al. found HSV-1 genome by using an in situ DNA–RNA hybridization method in the peripheral blood mononuclear cells of BD patients [3]. Based on these results, Sohn et al. [7] developed an animal model of BD by inoculating 1 × 10^6^ p.f.u. HSV type 1 (KOS strain) to needle-scratched earlobes of Institute of Cancer Research (ICR) mice, which are commonly used as an outbred population [10], and found that the mice developed BD-like symptoms, including genital ulcers, oral ulcers, skin lesions, eye lesions, arthritis, and intestinal ulcers [7]. These induced BD-like symptoms were similar to the clinical manifestations of BD in patients. Even though this mouse model was developed by inoculation with HSV, the disease pattern was very similar to that of human BD when evaluated in immune modulation experiments. HSV ribonucleotide reductase 1 mRNA was not detected in skin lesions of mice through polymerase chain reaction (PCR), even this lesion was induced by HSV inoculation [1]. This means the symptoms are not derived from HSV infection itself, but from dysregulated or uncontrolled immune responses triggered by HSV. HSV type 1 UL48 proteins were highly reactive to the serum of BD patients and BD mice [11]. In addition, in human herpesvirus 4, EBV (Epstein-Barr virus) shedding was increased in BD patients [12]. Innate and adaptive immune responses, including Th1 and Th2 imbalance, are thought to be responsible for managing HSV evading, and for maintaining surveillance during latency [13]. The pathogenic role of HSV related to immune dysregulation in BD is important, so it needs to be further clarified [14]. Many scientists developed animal models, including the HSV-induced model, for use in Behçet’s disease. They are listed in Table 1.

### 2.2. Genetic Susceptibility

Genetic susceptibility of BD has been widely studied. It is well-known that human leukocyte antigen (HLA)-B gene, particularly HLA-B51, is associated with BD patients [15]. However, the relative risk of BD associated with HLA-B51 varies widely among different ethnic populations. HLA-B51 allele is more frequent in some populations where BD is virtually unknown [16]. It has been recognized that the association of HLA-B51 with BD is stronger in Turkish and Japanese populations than in the Western population. Transgenic mice with HLA-B51 generated by Takeno et al. [17] have shown neutrophil hyperfunction without showing any clinical manifestations that mimic BD. This indicates the HLA-B51 molecule alone is insufficient to induce clinical BD. This result suggests that other factors are involved in BD susceptibility.

HLA-G gene variants have also been studied in BD [18,19]. HLA-G was first identified in placenta. It is involved in maintaining tolerance of maternal immune system to semi-allogeneic fetus [20,21,22]. HLA-G has an inhibitory effect on cytotoxicity of natural killer (NK) and T-cells, and T-cell proliferation [23]. HLA-G can inhibit trans-endothelial migration of NK cells [24], shift the cytokine balance toward Th2 dominance [25], and suppress the proliferation of allogeneic CD4+ T lymphocytes [26,27]. These results suggest that HLA-G has specific inhibitory effects on immune cells. Qa-2 is the functional homolog of HLA-G in mice [28]. HSV-induced BD mice show decreased Qa-2 levels. Down-regulation of Qa-2 is known to bring the deterioration of BD-like symptoms [29].

### 2.3. Immuological Dysregulation

HSV induced BD-like symptoms have been reported in mice [7]. However, virus infection alone is insufficient to explain the pathogenesis of BD. In BD patients, dysregulations of immune functions have been reported. There is plenty of evidence for aberrant T-cell responses in BD. Type 1 helper T cells (Th1) are known to secrete interleukin (IL)-2, and interferon gamma (IFNγ) activates macrophages, and elicits delayed hypersensitivity reactions, while type 2 helper T cells (Th2) can produce IL-4, IL-5, and IL-10 to suppress cell-mediated immunity. In BD, these cytokines perform a cross-regulatory function between Th1 and Th2 subsets. Th1/Th2 imbalance is thought to be an important factor in BD [30,31]. The induction of macrophages promotes Th1-dependent cellular immune responses and suppresses Th2 cell activity [32]. Sohn et al. [33] have demonstrated that deletion of macrophages decreases the incidence of BD in mice. Deletion of macrophages also up-regulates Th2 cytokines that are closely related to improvement of BD symptoms. Regulatory T (Treg) cells play an important role in the pathogenesis of autoimmune disorders, including BD. Frequencies of Treg cells are reduced in the peripheral blood of BD patients with eye lesions [34]. Therefore, low levels of CD4+CD25+ regulatory T cells can be a factor in the pathogenesis of BD. According to Shim et al. [35], when CD4+CD25+ T cells isolated from healthy mice are transferred to BD mice, the disease severity is significantly down-regulated by Treg cells transferred in a dose-dependent manner. Increased Treg cells up-regulated IL-10 and TGF-β levels, but down-regulated IFNγ, tumor necrosis factor alpha (TNFα), IL-6, and IL-17 levels.

## 3. Therapeutic Applications of Inflammatory Mice Model of Behçet’s Disease Induced by HSV

### 3.1. Natural Products

#### 3.1.1. TNFα Inhibition with Derivatives of *Gentiana Macrophylla* Radix

**Figure 1:**
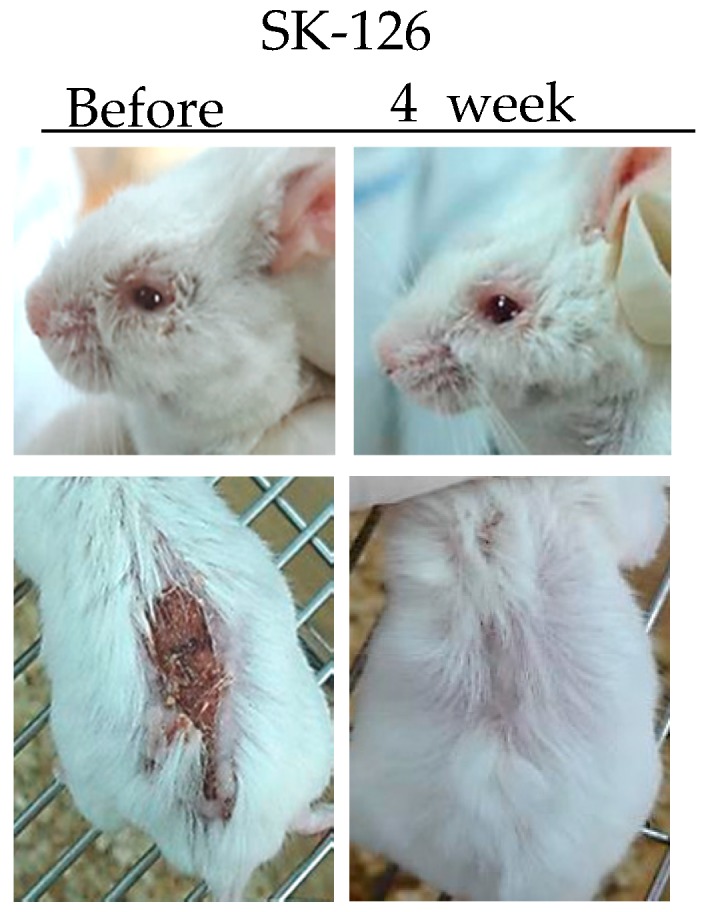
Changes in symptoms of herpes simplex virus (HSV)-induced Behçet’s disease mouse after treatment with SK126.

TNFα is a potent paracrine and endocrine mediator of inflammatory and immune functions. There is growing evidence showing that TNFα plays an important role in the management of inflammatory diseases, including BD. In BD patients, TNFα production is high [36]. Infliximab (anti-TNFα antibody) and Etanercept (soluble TNF receptor) have been used to treat BD patients [37,38]. Inhibition of TNFα expression by administration of TNFα small interfering RNA (siRNA) can ameliorate HSV-induced BD mice. The severity score of BD was significantly decreased compared to that in the control group [39]. SK126 and SK94 are synthesized pyridine derivatives based on gentianine, a major component of *Gentiana Macrophylla Radix.* When SK126 and SK94 were used to treat TNFα-stimulated human umbilical vein endothelial cells, they down-regulated adhesion molecules such as ICAM-1, VCAM-1, and E-selectin [40]. In addition, oral administration of SK126 and SK94 effectively down-regulated serum levels of TNFα in HSV-induced BD mice accompanied by symptom improvement [40] (Figure 1). 

#### 3.1.2. Herba Taraxaci

Herba Taraxaci (*Taraxacum mongolicum* Hand.-Mazz.) is frequently used for bacterial and viral infections. It has anti-inflammtory, anti-carcinogenic, anti-allergic, anti-hyperglycemic, and analgesic activities [41]. Its known effects include reducing heat, decreasing edema, and clearing toxic substances in inflamed areas. In addition, it has antibiotic effects against *Staphylococcus aureus*, *Streptococcus agalactiae*, and *Streptococcus dysgalactiae* [42]. Herba Taraxaci contains taraxasterol, choline, inulin, and pectin [43]. Treatment of BD mice with Herba Taraxaci can induce IL-4 and improve symptoms. Herba Taraxaci can alter Th1/Th2 balance by increasing IL-4 and Th2 immune responses [33,43]. In addition, Herba Taraxaci can induce Fas-mediated apoptosis of abnormal proliferating leukocytes involved in the induction of BD symptoms [43]. Nakamura et al. have suggested that activated CD4+ T cells may cause severe chronic inflammation due to the insufficient expression of Fas in BD patients [44]. A combination therapy of Herba Taraxaci and colchicine can reduce symptoms in 80% of BD mice, much higher than colchicine (30%) or famciclovir (40%) treatment alone. Herba Taraxaci alone or in combination with colchicine can up-regulate frequencies of IL-10 secreting splenocytes. Increased IL-10 by Herba Taraxaci might have acted as an improvement factor. Thus, combination therapy with natural products can be another strategy for BD treatment.

#### 3.1.3. Chitosan

Chitosan is a biocompatible, biodegradable, and nontoxic natural polymer with high cationic potential [45]. Chitosan is a safe and effective adjuvant with many biological effects on drug delivery [46]. A mixture of chitosan and pCIN-mIL-4 DNA vector can significantly increase IL-4 mRNA and IL-4 protein levels after in vivo mouse administration [47]. Oral administration of pCIN-mIL-4 DNA vector in combination with chitosan has effectively delivered DNA vector to intestinal tissues of mice. Our recent study has shown that chitosan itself can be a potential immune modulator [48]. Oral administration of chitosan significantly up-regulated frequencies of DX5+ natural killer cell populations in peripheral blood leukocytes (PBL). In HSV-infected mice, chitosan increased the frequencies of CD4+ T cells, CD8+ T cells, and CD11c+ dendritic cells in PBL. In addition, chitosan treatment down-regulated the levels of anti-HSV antibodies in the serum of HSV-infected mice, compared to the control group [48]. Thus, chitosan can be used as an adjuvant for gene delivery and an immune modulator by oral administration. 

### 3.2. DNA Vector

#### 3.2.1. Interleukin-4

Macrophages are phenotypically heterogeneous. They perform various activities in parallel with adaptive immune responses of Th1 and Th2. M1 macrophages are activated in response to endogenous or exogenous inflammatory stimuli such as Th1 cytokine IFNγ [49] while M2 macrophages are activated by Th2 cytokines such as IL-4 or IL-13 [50]. During the chronic stage of infection, macrophages are further activated by cytokines secreted by T cells. Increased IL-4 may induce the apoptosis of IFNγ-producing macrophages [51]. According to the counter-regulation model of Th1 and Th2 cytokines, an increase of IL-4 might decrease IFNγ producing macrophages in BD mice, or induce apoptosis of IFNγ-secreting macrophages [51,52]. Treatment of recombinant IL-4 can significantly reduce the M1/M2 ratio in mice, and alleviate BD symptoms by downregulating IL-17 and IL-8 [53]. Subcutaneous injection of the IL-4-expressing vector by gene gun bombardment also improves BD symptoms in mice by enhancing serum IL-4, which alters the Th1 response toward the Th2 response [51]. Increase of mRNA and protein levels of IL-4 were also observed after the administration of a mixture of chitosan and pCIN-mIL-4 in mice [47].

#### 3.2.2. C-C Chemokine Ligand 21

Chemokine ligand 21 (CCL21) is a C-C chemokine family produced in the reticular cells of secondary lymphoid organs. CCL21 binds to its receptor CCR7. The level of CCL21 is lower in the synovial fluid of BD patients. It has been suggested that a lack of CCL21 is associated with inflammation [54,55]. HSV-1-induced BD symptomatic mice have also shown a lower expression of CCL21 compared to asymptomatic mice. Transfection of pcDNA3.1-CCL21 DNA vector can increase CCL21 protein expression in RAW 264.7 cells (macrophage like, Abelson leukemia virus transformed cell line) while intraperitoneal injection of pcDNA3.1-CCL21 can increase the frequencies of CCR7+ PBL in normal mice and BD mice [56]. Expression of CCL21 is also associated with the up-regulation of CD8+CD44+ memory T cells and CD8+CD62L-memory T cells. In BD patients, the frequencies of CD4+CD45RO+ memory T cells are increased after symptom improvement [57,58]. Therefore, the use of CCL21 can help improve BD by regulating CCR7+ cells and memory T cells.

### 3.3. RNA and siRNA

#### 3.3.1. Polyinosinic:Polycytidylic Acid (Poly I:C)

Polyinosinic:polycytidylic acid (Poly I:C) is a mismatched double-stranded RNA. One strand is a polymer of inosinic acid, while the other strand is a polymer of cytidylic acid. Poly I:C induces IL-15 and IL-15 receptor alpha (IL-15Rα) and stimulates the production of memory T cells [59,60,61]. Poly I:C administration can increase IL-15Rα in normal mice and HSV-induced BD mice, leading to ameliorated BD symptoms [62]. Our previous study has confirmed that CD8+CD44+ and CD8+CD62L- memory T cells are significantly lower in BD mice. Poly I:C application can significantly increase CD4+CD44+ memory T cells and CD8+ central memory T cells in BD mice. It has been reported that the long-term survival of CD8+ memory T cells is correlated with an improvement in BD symptoms [56,62,63]. Poly I:C may affect the long-term survival of memory T cells. It can increase the frequencies of memory T cells. In addition, Poly I:C can up-regulate IL-10, a potent anti-inflammtory cytokine, in BD mice. Increase of IL-10 level has been found to be accompanied by lower IL-23R mRNA and IL-17A protein levels [62]. Taken together, these findings suggest that Poly I:C might have therapeutic application in BD treatment.

#### 3.3.2. TNFα siRNA

TNFα is an effective paracrine and endocrine inflammatory mediator. It can transmit signals between the immune system and other cells [39,64]. In several acute and chronic inflammatory diseases such as inflammatory bowel disease [65], Crohn’s disease [64], Rheumatoid arthritis [66], atopic dermatitis [67], and Behçet’s disease [36], overexpression of TNFα has been observed. It has been published anti-TNFα antibody therapy can improve BD symptoms [68,69]. siRNA intraperitoneal treatment can reduce the overexpression of TNFα in the serum of BD mouse. siRNA binds RNA-Induced Silencing Complex (RISC), a multiprotein component complex in cytoplasm, and then the passenger strand of siRNA departs, thereby commencing the RNA interference process via target mRNA cleavage, and degradation results [70]. Such decrease in the level of TNFα can improve HSV-induced BD symptoms in mice [39]. Infliximab is now widely used in the management of autoimmune diseases including BD. Infliximab blocks the action of TNFα by binding to it and preventing TNFα from binding to signaling receptors on the cell surface. TNFα siRNA can act faster than infliximab. Thus, it is more effective than infliximab in BD mice [39]. TNFα siRNA has shown therapeutic efficacy in HSV-induced BD mice, supporting that RNAi therapeutics or gene silencing can be a potential new class of drug for managing inflammatory diseases, including BD.

#### 3.3.3. Interleukin-6 siRNA

**Figure 2:**
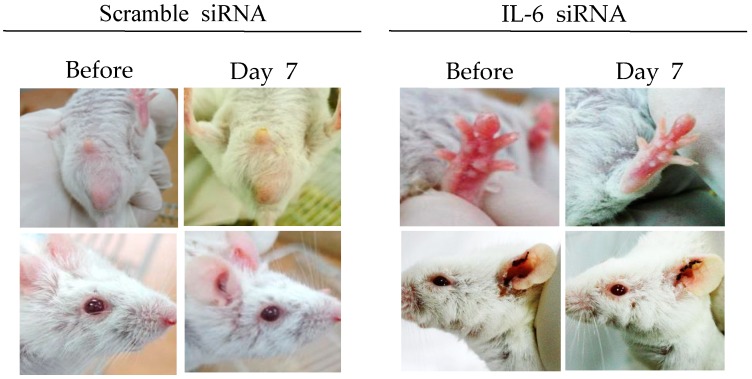
IL-6 small interfering RNA (siRNA) improves HSV-induced Behçet’s disease symptoms. IL-6 siRNA (1.5 µg in 200 µL of 5% glucose solution) was intraperitoneally injected twice at 3-day intervals.

IL-6 is a multifunctional cytokine secreted by lymphoid and non-lymphoid cells. It is involved in the regulation of immune responses and inflammation [71,72]. HSV type 1 and type 2 are potent inducers for IL-6. IL-6 is released at a relatively early stage following HSV infection [73,74]. IL-6 levels are significantly higher in BD patients and HSV-induced BD mice, indicating that IL-6 plays a pathogenic role in BD [75,76]. It has been reported that treatment with IL-6 siRNA can reduce serum IL-6 levels and decrease expression of RORγt (retinoic acid-receptor-related orphan nuclear receptor gamma t), and TNFα in mice [75]. IL-6 siRNA treatment can also increase Treg cells and improve BD symptoms, such as oral ulcers, scrotal inflammation, arthritis, and skin ulcers (Figure 2) [35,75]. IL-6 siRNA treatment can also reduce IL-17 and IL-23p40 (alpha-receptor subunit of IL-23 and IL-12) levels in the sera of BD mice [75]. Therefore, IL-6 siRNA can be used as a therapeutic to decrease IL-6 levels in BD patients. 

#### 3.3.4. miRNA-21 Antagomir

**Figure 3:**
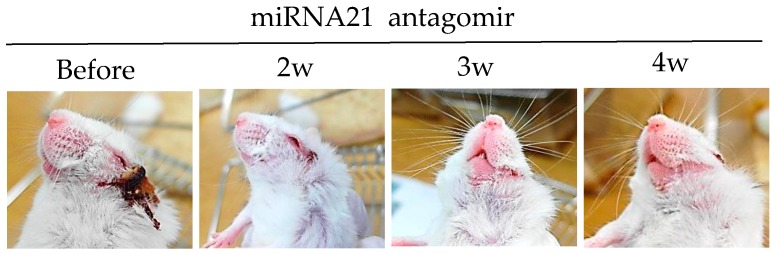
miRNA-21 inhibition with miRNA-21 antagomir improves HSV induced Behçet’s disease symptom.

MicroRNAs (miRNAs) are small non-coding RNAs that play critical roles in immune functions [77]. Single miRNA also has a substantial impact on immune regulation. Decreased miRNA-155 levels are associated with ocular BD patients [78]. miRNA-146a gene polymorphisms have also been found in ocular BD patients [79]. miRNA-21 is frequently up-regulated in solid tumors [80]. Overexpression of miRNA-21 in cancer promotes cell survival and reduces apoptosis. miRNA-21 is one the most abundant miRNAs in T cells, especially in effector T cells. Its expression is dynamically changed during T cell differentiation [81,82]. miRNA-21 is highly expressed in BD patients and HSV-induced BD mice [83]. Intraperitoneal treatment of miRNA-21 antagomirs targeting the host miRNA-21, can improve BD symptoms in mice with the downregulation of IL-17, IL-6, and TLR-4 [83]. Choi et al. [83] have clearly shown that miRNA-21 expression is correlated with BD symptoms in both patients and mice (Figure 3).

### 3.4. Protein Complex

#### 3.4.1. IL-2/IL-2 Antibody Complex

Serum levels of IL-2 in BD patients are controversial. Some studies have reported that IL-2 serum levels are significantly higher in BD patients, with frequencies of IL-2 producing CD4+ and CD8+ cells being higher in active BD patients than those in inactive BD patients [71,84]. On the other hand, other studies have shown that IL-2 levels are not significantly different between BD patients and healthy controls, although soluble interleukin 2 receptor (sIL-2R) is elevated in active BD patients, suggesting that sIL-2R is related to disease activity [85]. IL-2R is composed of alpha (CD25), beta (CD122), and gamma (CD132) subunits. It has been reported that IL-2Rβ levels are significantly lower in BD patients, and HSV induced BD mice [86]. Treatment with IL-2/S4B6-1 (IL-2/anti-mIL-2 antibody complex) can significantly upregulate IL-2Rα, IL-2Rβ, IL-2Rγ, and regulatory T cells. IL-2/S4B6-1 also regulates NK cell maturation in normal mice and HSV-induced BD mice [86]. Elevated IL-2Rβ is associated with increased proportions of central memory T cells. Up-regulation of central memory T cells is correlated with inhibition of BD deterioration in mice [56,87]. Likewise, IL-2Rβ blocking with anti-IL-2Rβ antibody injection exacerbates BD symptoms with down-regulated frequencies of IL-2Rβ+ cells in mice [86]. Choi et al. [86] have shown a correlation between IL-2R subunit expressing cells and BD in both patients and mice.

#### 3.4.2. IL-15/IL-15Rα Complex

IL-15 is a pleiotropic cytokine involved in the pathogenesis of diverse inflammatory diseases, including BD. IL-15 is highly expressed in the serum, cerebral fluids, and ocular fluids of BD patients, whereas IL-15Rα expressing cells are lower in the peripheral blood of BD patients [88]. IL-15Rα restricts aberrant immune stimulation and decreases the risk of uncontrolled IL-15 exposure [89]. Recently, it has been shown that an IL-15/IL-15Rα complex expressing the vector or the recombinant mIL-15/IL-15Rα-Fc protein complex can decrease disease severity and ameliorate symptoms in HSV-induced BD mice [90]. According to a previous report, frequencies of Treg cells are down-regulated in BD mice [35]. rmIL-15/IL-15Rα–Fc treatment can increase the frequencies of Treg cells that can attenuate BD symptoms [90]. In addition, treatment with anti-TNFα antibody, infliximab, can increase IL-15Rα+ cell frequencies. Therefore, IL-15/IL-15Rα complex can be used as a therapeutic candidate for BD.

### 3.5. Vitamin & Galactose Derivatives

#### 3.5.1. Vitamin D3

TLR-2 and TLR-4 are expressed in mouse and human macrophages and monocytes. Accumulated TLR-2 and TLR-4 expressing cells have been found in lesions of BD patients [91]. TLR-2 expressing cells play a pivotal role in initiating destructive Th1-type responses at the site of BD lesions [92]. This suggests that the regulation of TLR-2 and TLR-4 might be involved in BD pathogenesis. Vitamin D reduction is associated with increased renal inflammation and BD. Vitamin D3 treatment can reduce chemokine synthesis and monocyte trafficking, and downregulate TLR-2 and TLR-4 on mononuclear cells [93,94,95]. Treatment with vitamin D3 (1,25(OH)_2_D_3_) can also down-regulate TLR-2 and TLR-4 in the peritoneal macrophages of BD mice in a dose-dependent manner, leading to reduced severity scores [96]. Serum levels of IL-6 and TNFα are also down-regulated after treatment with 1,25(OH)_2_D_3_ in BD mice [96]. These results suggest that 1,25(OH)_2_D_3_ can be used as a complementary treatment for BD.

#### 3.5.2. N-Acetylgalactosamine (GalNAc), a Mannose Receptor Ligand

CD206 is a mannose receptor that is mainly expressed on macrophages. It is involved in various autoimmune diseases. Frequencies of CD206 positive cells are higher in whole leukocytes and monocytes of active BD patients than in those of inactive BD patients. This indicates that CD206 is involved in the pathogenesis of BD [97]. CD206 can bind to HSV-1 through macrophage and dendritic cells. HSV-2 infected patients show enhanced prevalence of CD206 expressing dendritic cells [98]. CD206 has elevated frequencies in HSV-1 induced BD mice [99]. Colchicine and pentoxifylline are commercial drugs used for BD patients. They can decrease frequencies of CD206 in HSV-1-induced BD mice, accompanied by down-regulation of IL-17. Treatment with 4-sulfated N-acetyl galactosamine (GalNAc), a ligand of CD206, can significantly decrease CD206 in BD mice. It can also reduce the levels of IL-17 [99]. These results suggest that the inhibition of CD206 may provide therapeutic benefits for BD patients.

## 4. Conclusions

**Figure 4:**
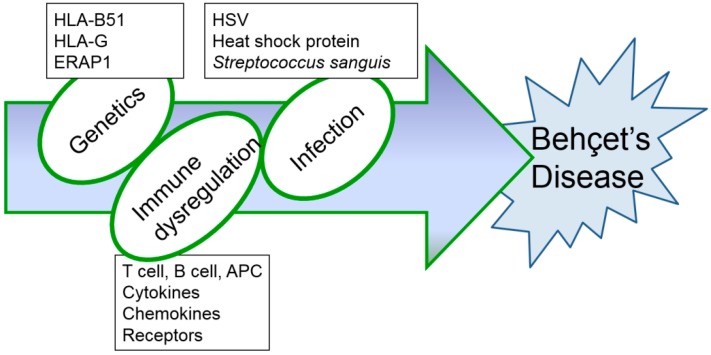
Hypothesized etiopathogenetic scheme of Behçet’s disease.

Although several immunological abnormalities have been demonstrated, the precise mechanism of inflammatory changes in BD has not yet been completely determined. Up to date, HSV-induced BD mouse models have been consistently used (Table 1 and Table 2) as they produce symptoms that are most similar to those seen in patients with BD. Further research is needed to find the most appropriate therapy to overcome this rare, intractable disease. Based on results obtained from BD mice induced by HSV, successful treatment strategies for BD are expected to be developed in the near future. In addition, HSV inoculation alone is not sufficient to develop BD models. HSV inoculation to HLA B51 transgenic mice [17] may increase BD-like phenotypes and can be a potent animal model for BD. Erap1 (Endoplasmic reticulum aminopeptidase 1) is also a candidate gene for BD [100]. HSV inoculation to Erap1 deficient mice also can be a more advanced animal model for BD. Management of BD is still unsatisfactory because of its complex entities (Figure 4). Further study in advanced animal models will be significant progress for the BD research field.

## Figures and Tables

**Table 1:** Published animal models of Behçet’s disease (1979–2018).

Models and Symptoms	Published Number of Papers
Environmental pollutants→Mucocutaneous lesions in miniature swine	1
Human 60 kD heat shock protein-derived peptide 336–351→Uveitis in rats	1
HLA-B51 Transgenic mice→Excessive function of peripheral blood neutrophils but no symptoms	1
Herpes simplex virus type 1→Oral, genital, and skin ulcers, eye involvement, arthritis, and intestinal involvement in ICR mice and C57BL/6 mice	26
Tropomyosin→Inflammation in the skin, joints, and eyes of Lewis rats	2
Retinal soluble antigen (S-Ag)→Uveitis in rats	1
Sera from seven NeuroBD patients→Reduced locomotor activity in rats	1

**Table 2:** Applied therapeutic modalities for HSV-induced Behçet’s disease mice.

Categories	Applied Materials	Therapy Targets
**Conventional therapies**	Thalidomide Infliximab Etanercept	↓TNFα ↓TNFα ↓TNFα
**Natural products**	Derivatives of Gentiana macrophylla Radix Taraxacum mongolicum Hand.-Mazz. Chitosan	↓TNFα ↑IL-10 ↑IL-4
**Potential therapies**	IL-4 C-C chemokine ligand 21 Poly I:C TNFα siRNA IL-6 siRNA miRNA-21 antagomir IL-2/IL-2 Ab complex, IL-15/IL-15R-Fc complex Vitamin D3 4-sulfated N-acetyl galactosamine	↑IL-4 ↑CCR7 ↑IL-15Rα, IL-10 ↓TNFα ↓IL-6 ↓miRNA-21 ↑IL-2Rα, ↑IL-15Rα ↓TLR-2, ↓TLR-4, ↓CD206

Note: IL, Interleukin; ↓, Downregulation; ↑, Upregulation.

## References

[1] Sohn S., Lee E.S., Bang D. (2012). Learning from HSV-infected mice as a model of Behcet’s disease. Clin. Exp. Rheumatol..

[2] Greco A., De Virgilio A., Ralli M., Ciofalo A., Mancini P., Attanasio G., de Vincentiis M., Lambiase A. (2018). Behçet’s disease: New insights into pathophysiology, clinical features and treatment options. Autoimmun. Rev..

[3] Eglin R.P., Lehner T., Subak-Sharpe J.H. (1982). Detection of RNA complementary to herpes-simplex virus in mononuclear cells from patients with Behcet’s syndrome and recurrent oral ulcers. Lancet.

[4] Evans A.D., Pallis C.A., Spillane J.D. (1957). Involvement of the nervous system in Behcet’s syndrome; report of three cases and isolation of virus. Lancet.

[5] Sezer F.N. (1953). The isolation of a virus as the cause of behçet’s disease. Am. J. Ophthalmol..

[6] Studd M., McCance D.J., Lehner T. (1991). Detection of HSV-1 DNA in patients with Behcet’s syndrome and in patients with recurrent oral ulcers by the polymerase chain reaction. J. Med. Microbiol..

[7] Sohn S., Lee E.S., Bang D., Lee S. (1998). Behcet’s disease-like symptoms induced by the herpes simplex virus in ICR mice. Eur. J. Dermatol..

[8] Lee S., Bang D., Cho Y.H., Lee E.S., Sohn S. (1996). Polymerase chain reaction reveals herpes simplex virus DNA in saliva of patients with Behcet’s disease. Arch. Dermatol. Res..

[9] Tojo M., Zheng X., Yanagihori H., Oyama N., Takahashi K., Nakamura K., Kaneko F. (2003). Detection of herpes virus genomes in skin lesions from patients with Behcet’s disease and other related inflammatory diseases. Acta Derm. Venereol..

[10] Kim J.E., Nam J.H., Cho J.Y., Kim K.S., Hwang D.Y. (2017). Annual tendency of research papers used ICR mice as experimental animals in biomedical research fields. Lab. Anim. Res..

[11] Zheng Z., Sohn S., Ahn K.J., Bang D., Cho S.B. (2015). Serum reactivity against herpes simplex virus type 1 UL48 protein in Behcet’s disease patients and a Behcet’s disease-like mouse model. Acta Derm. Venereol..

[12] Seoudi N., Bergmeier L.A., Hagi-Pavli E., Bibby D., Fortune F. (2015). The seroprevalence and salivary shedding of herpesviruses in Behcet’s syndrome and recurrent aphthous stomatitis. J. Oral. Microbiol..

[13] Paludan S.R., Bowie A.G., Horan K.A., Fitzgerald K.A. (2011). Recognition of herpesviruses by the innate immune system. Nat. Rev. Immunol..

[14] Kim D.Y., Cho S., Choi M.J., Sohn S., Lee E.S., Bang D. (2013). Immunopathogenic role of herpes simplex virus in Behcet’s disease. Genet. Res. Int..

[15] Ohno S., Ohguchi M., Hirose S., Matsuda H., Wakisaka A., Aizawa M. (1982). Close association of HLA-Bw51 with Behcet’s disease. Arch. Ophthalmol..

[16] Verity D.H., Marr J.E., Ohno S., Wallace G.R., Stanford M.R. (1999). Behcet’s disease, the silk road and HLA-B51: Historical and geographical perspectives. Tissue Antigens..

[17] Takeno M., Kariyone A., Yamashita N., Takiguchi M., Mizushima Y., Kaneoka H., Sakane T. (1995). Excessive function of peripheral blood neutrophils from patients with Behcet’s disease and from HLA-B51 transgenic mice. Arthritis Rheum..

[18] Park K.S., Nam J.H., Lee E.S., Choi J.S., Bang D., Lee S. (2006). Increased risk of human leukocyte antigen-G gene variants in Behçet’s disease. Clin. Exp. Rheumatol..

[19] Park K.S., Park J.S., Nam J.H., Bang D., Sohn S., Lee E.S. (2007). HLA-E*0101 and HLA-G*010101 reduce the risk of Behcet’s disease. Tissue Antigens..

[20] Kovats S., Main E.K., Librach C., Stubblebine M., Fisher S.J., DeMars R. (1990). A class I antigen, HLA-G, expressed in human trophoblasts. Science.

[21] McMaster M.T., Librach C.L., Zhou Y., Lim K.H., Janatpour M.J., DeMars R., Kovats S., Damsky C., Fisher S.J. (1995). Human placental HLA-G expression is restricted to differentiated cytotrophoblasts. J. Immunol..

[22] Michita R.T., Zambra F.M.B., Fraga L.R., Sanseverino M.T.V., Callegari-Jacques S.M., Vianna P., Chies J.A.B. (2016). A tug-of-war between tolerance and rejection–new evidence for 3′UTR HLA-G haplotypes influence in recurrent pregnancy loss. Hum. Immunol..

[23] Riteau B., Rouas-Freiss N., Menier C., Paul P., Dausset J., Carosella E.D. (2001). HLA-G2, -G3, and -G4 isoforms expressed as nonmature cell surface glycoproteins inhibit nk and antigen-specific CTL cytolysis. J. Immunol..

[24] Dorling A., Monk N.J., Lechler R.I. (2000). HLA-G inhibits the transendothelial migration of human NK cells. Eur. J. Immunol..

[25] Kanai T., Fujii T., Unno N., Yamashita T., Hyodo H., Miki A., Hamai Y., Kozuma S., Taketani Y. (2001). Human leukocyte antigen-G-expressing cells differently modulate the release of cytokines from mononuclear cells present in the decidua versus peripheral blood. Am. J. Reprod. Immunol..

[26] Bainbridge D.R., Ellis S.A., Sargent I.L. (2000). HLA-G suppresses proliferation of CD4(+) T-lymphocytes. J. Reprod. Immunol..

[27] Riteau B., Menier C., Khalil-Daher I., Sedlik C., Dausset J., Rouas-Freiss N., Carosella E.D. (1999). HLA-G inhibits the allogeneic proliferative response. J. Reprod. Immunol..

[28] Comiskey M., Goldstein C.Y., De Fazio S.R., Mammolenti M., Newmark J.A., Warner C.M. (2003). Evidence that HLA-G is the functional homolog of mouse Qa-2, the ped gene product. Hum. Immunol..

[29] Lee M., Choi B., Kwon H.J., Shim J.A., Park K.S., Lee E.S., Sohn S. (2010). The role of Qa-2, the functional homolog of Hla-G, in a Behcet’s disease-like mouse model induced by the herpes virus simplex. J. Inflamm..

[30] Frassanito M.A., Dammacco R., Cafforio P., Dammacco F. (1999). Th1 polarization of the immune response in Behcet’s disease: A putative pathogenetic role of interleukin-12. Arthritis Rheum..

[31] Raziuddin S., al-Dalaan A., Bahabri S., Siraj A.K., al-Sedairy S. (1998). Divergent cytokine production profile in Behcet’s disease. Altered Th1/Th2 cell cytokine pattern. J. Rheumatol..

[32] Desmedt M., Rottiers P., Dooms H., Fiers W., Grooten J. (1998). Macrophages induce cellular immunity by activating Th1 cell responses and suppressing Th2 cell responses. J. Immunol..

[33] Sohn S., Lee E.-S., Kwon H.J., Lee S.I., Bang D., Lee S. (2001). Expression of Th2 cytokines decreases the development of and improves Behçet’s disease–like symptoms induced by herpes simplex virus in mice. J. Infect. Dis..

[34] Nanke Y., Kotake S., Goto M., Ujihara H., Matsubara M., Kamatani N. (2008). Decreased percentages of regulatory T cells in peripheral blood of patients with Behcet’s disease before ocular attack: A possible predictive marker of ocular attack. Mod. Rheumatol..

[35] Shim J., Lee E.S., Park S., Bang D., Sohn S. (2011). CD4(+) CD25(+) regulatory T cells ameliorate Behcet’s disease-like symptoms in a mouse model. Cytotherapy.

[36] El Menyawi M., Fawzy M., Al-Nahas Z., Edris A., Hussein H., Shaker O., Elwan H. (2014). Serum tumor necrosis factor alpha (TNF-α) level in patients with Behçet’s disease: Relation to clinical manifestations and disease activity. Egypt. Rheumatol..

[37] Mohammed R.H. (2014). Etanercept therapy in Behcet’s disease. The tight control strategy in refractory disease. Z. Rheumatol..

[38] Ugras M., Ertem D., Celikel C., Pehlivanoglu E. (2008). Infliximab as an alternative treatment for Behçet disease when other therapies fail. J. Pediatr. Gastroenterol. Nutr..

[39] Choi B., Hwang Y., Kwon H.J., Lee E.S., Park K.S., Bang D., Lee S., Sohn S. (2008). Tumor necrosis factor alpha small interfering RNA decreases herpes simplex virus-induced inflammation in a mouse model. J. Dermatol. Sci..

[40] Choi B., Kim J., Lee E.S., Bang D., Sohn S. (2011). Synthesized pyridine compound derivatives decreased TNF alpha and adhesion molecules and ameliorated HSV-induced inflammation in a mouse model. Eur. J. Pharmacol..

[41] Schütz K., Carle R., Schieber A. (2006). Taraxacum—A review on its phytochemical and pharmacological profile. J. Ethnopharmacol..

[42] Sun S., Dai W., Yu H., Wang Y., Wang X., Peng S. (2015). Antibacterial activity of aqueous and ethanolic extracts of Portulaca oleracea L. And Taraxacum mongolicum Hand.-Mazz against pathogenic bacteria of cow mastitis. Indian J. Anim. Res..

[43] Sohn S., Bang D., Lee S.I., Kim Y.A., Lee E.S., Ha J.Y., Kim J.H., Choi S.Y., Lee S. (2003). Combined treatment with colchicine and Herba Taraxaci (Tarazacum mongolicum Hand.-Mazz.) attenuates Behcet’s disease-like symptoms in mice and influences the expressions of cytokines. Int. Immunopharmacol..

[44] Nakamura S., Sugita M., Matoba H., Tanaka S., Isoda F., Ohno S. (1996). Insufficient expression of Fas antigen on helper t cells in behcet’s disease. Br. J. Ophthalmol..

[45] Carlos P., Waldo A.M., Hazel P., Niuris A. (2003). Chitosan: An attractive biocompatible polymer for microencapsulation. Macromol. Biosci..

[46] Sailaja A.K., Amareshwar P., Chakravarty P. (2010). Chitosan nanoparticles as a drug delivery system. Res. J. Pharm. Biol. Chem. Sci..

[47] Choi B.C., Choi J.Y., Sohn S., Amexcua-Guerra L.M. (2011). Oral delivery of DNA vector conjugated with chitosan and its effect on Th1 polarized inflammation. Advances in the Diagnosis and Treatemnt of Vasculitis.

[48] Choi B., Jo D.H., Anower A.K., Islam S.M., Sohn S. (2016). Chitosan as an immunomodulating adjuvant on T-cells and antigen-presenting cells in herpes simplex virus type 1 infection. Mediators Inflamm..

[49] Gratchev A., Kzhyshkowska J., Kothe K., Muller-Molinet I., Kannookadan S., Utikal J., Goerdt S. (2006). Mphi1 and Mphi2 can be re-polarized by Th2 or Th1 cytokines, respectively, and respond to exogenous danger signals. Immunobiology.

[50] Stout R.D., Suttles J. (2004). Functional plasticity of macrophages: Reversible adaptation to changing microenvironments. J. Leukoc. Biol..

[51] Lee S.I., Kwon H.J., Lee E.S., Yang B.C., Bang D., Lee S., Sohn S. (2007). Using pcin-mil-4 DNA vector to express mrna and protein and to improve herpes simplex virus-induced Behcet’s disease symptoms in mice. Vaccine.

[52] Miyashita H., Katayama N., Fujieda A., Shibasaki T., Yamamura K., Sugimoto Y., Miyata E., Ohishi K., Nishii K., Masuya M. (2005). IL-4 and IL-10 synergistically inhibit survival of human blood monocytes supported by GM-CSF. Int. J. Oncol..

[53] Anower A.K., Shim J.A., Choi B., Kwon H.J., Sohn S. (2014). The role of classical and alternative macrophages in the immunopathogenesis of herpes simplex virus-induced inflammation in a mouse model. J. Dermatol. Sci..

[54] Forster R., Davalos-Misslitz A.C., Rot A. (2008). Ccr7 and its ligands: Balancing immunity and tolerance. Nat. Rev. Immunol..

[55] Pay S., Musabak U., Simsek I., Erdem H., Pekel A., Sengul A., Dinc A. (2007). Synovial lymphoid neogenetic factors in Behcet’s synovitis: Do they play a role in self-limiting and subacute course of arthritis?. Clin. Exp. Rheumatol..

[56] Choi B., Lim H.C., Lee E.S., Anower A.K., Sohn S. (2013). CCL21 attenuates HSV-induced inflammation through up-regulation of CD8+ memory cells. Immunobiology.

[57] Direskeneli H., Ergun T., Yavuz S., Hamuryudan V., Eksioglu-Demiralp E. (2008). Thalidomide has both anti-inflammatory and regulatory effects in Behcet’s disease. Clin. Rheumatol..

[58] Jalili A., Pashenkov M., Kriehuber E., Wagner C., Nakano H., Stingl G., Wagner S.N. (2010). Induction of targeted cell migration by cutaneous administration of a DNA vector encoding a biologically active chemokine CCL21. J. Invest. Dermatol..

[59] Lodolce J.P., Burkett P.R., Boone D.L., Chien M., Ma A. (2001). T cell-independent interleukin 15ralpha signals are required for bystander proliferation. J. Exp. Med..

[60] Lorenzen I., Dingley A.J., Jacques Y., Grotzinger J. (2006). The structure of the interleukin-15 alpha receptor and its implications for ligand binding. J. Biol. Chem..

[61] Wang Y., Cella M., Gilfillan S., Colonna M. (2010). Cutting edge: Polyinosinic:Polycytidylic acid boosts the generation of memory CD8 T cells through melanoma differentiation-associated protein 5 expressed in stromal cells. J. Immunol..

[62] Choi J., Lee E.S., Choi B., Sohn S. (2013). Therapeutic potency of Poly I:C in HSV-induced inflammation through up-regulation of IL-15 receptor alpha. Immunobiology.

[63] Sato N., Patel H.J., Waldmann T.A., Tagaya Y. (2007). The IL-15/IL-15ralpha on cell surfaces enables sustained IL-15 activity and contributes to the long survival of CD8 memory T cells. Proc. Natl. Acad. Sci. USA.

[64] Van Deventer S.J. (1997). Tumour necrosis factor and Crohn’s disease. Gut.

[65] Maeda M., Watanabe N., Neda H., Yamauchi N., Okamoto T., Sasaki H., Tsuji Y., Akiyama S., Tsuji N., Niitsu Y. (1992). Serum tumor necrosis factor activity in inflammatory bowel disease. Immunopharmacol. Immunotoxicol..

[66] Yamanaka H. (2015). TNF as a target of inflammation in rheumatoid arthritis. Endocr. Metab. Immune Disord. Drug Targets.

[67] Sumimoto S., Kawai M., Kasajima Y., Hamamoto T. (1992). Increased plasma tumour necrosis factor-alpha concentration in atopic dermatitis. Arch. Dis. Child..

[68] Karube H., Kamoi K., Ohno-Matsui K. (2016). Anti-TNF therapy in the management of ocular attacks in an elderly patient with long-standing Behçet’s disease. Int. Med. Case Rep. J..

[69] Desbois A.C., Addimanda O., Bertrand A., Deroux A., Pérard L., Depaz R., Hachulla E., Lambert M., Launay D., Subran B. (2016). Efficacy of Anti-TNFα in Severe and Refractory Neuro-Behcet Disease: An Observational Study. Medicine.

[70] Rao D.D., Vorhies J.S., Senzer N., Nemunaitis J. (2009). siRNA vs. shRNA: Similarities and differences. Adv. Drug Deliv. Rev..

[71] Akdeniz N., Esrefoglu M., Keles M.S., Karakuzu A., Atasoy M. (2004). Serum interleukin-2, interleukin-6, tumour necrosis factor-alpha and nitric oxide levels in patients with Behcet’s disease. Ann. Acad. Med. Singap..

[72] Heinrich P.C., Castell J.V., Andus T. (1990). Interleukin-6 and the acute phase response. Biochem. J..

[73] Kanangat S., Babu J.S., Knipe D.M., Rouse B.T. (1996). HSV-1-mediated modulation of cytokine gene expression in a permissive cell line: Selective upregulation of IL-6 gene expression. Virology.

[74] Paludan S.R. (2001). Requirements for the induction of interleukin-6 by herpes simplex virus-infected leukocytes. J. Virol..

[75] Shim J., Byun H.O., Lee Y.D., Lee E.S., Sohn S. (2009). Interleukin-6 small interfering rna improved the herpes simplex virus-induced systemic inflammation in vivo Behcet’s disease-like mouse model. Gene Ther..

[76] Yamakawa Y., Sugita Y., Nagatani T., Takahashi S., Yamakawa T., Tanaka S., Nakamura S., Ohno S., Sekihara H., Okuda K. (1996). Interleukin-6 (IL-6) in patients with Behcet’s disease. J. Dermatol. Sci..

[77] Davidson-Moncada J., Papavasiliou F.N., Tam W. (2010). miRNAs of the immune system: Roles in inflammation and cancer. Ann. N. Y. Acad. Sci..

[78] Zhou Q., Xiao X., Wang C., Zhang X., Li F., Zhou Y., Kijlstra A., Yang P. (2012). Decreased microRNA-155 expression in ocular Behcet’s disease but not in Vogt Koyanagi Harada syndrome. Invest. Ophthalmol. Vis. Sci..

[79] Zhou Q., Hou S., Liang L., Li X., Tan X., Wei L., Lei B., Kijlstra A., Yang P. (2014). microRNA-146a and Ets-1 gene polymorphisms in ocular Behcet’s disease and Vogt-Koyanagi-Harada syndrome. Ann. Rheum. Dis..

[80] Kumar S., Keerthana R., Pazhanimuthu A., Perumal P. (2013). Overexpression of circulating miRNA-21 and miRNA-146a in plasma samples of breast cancer patients. Indian J. Biochem. Biophys..

[81] Wang X., Tang S., Le S.Y., Lu R., Rader J.S., Meyers C., Zheng Z.M. (2008). Aberrant expression of oncogenic and tumor-suppressive micrornas in cervical cancer is required for cancer cell growth. PLoS ONE.

[82] Wu H., Neilson J.R., Kumar P., Manocha M., Shankar P., Sharp P.A., Manjunath N. (2007). miRNA profiling of naive, effector and memory CD8 T cells. PLoS ONE.

[83] Choi B., Kim H.A., Suh C.H., Byun H.O., Jung J.Y., Sohn S. (2015). The relevance of miRNA-21 in HSV-induced inflammation in a mouse model. Int. J. Mol. Sci..

[84] Sugi-Ikai N., Nakazawa M., Nakamura S., Ohno S., Minami M. (1998). Increased frequencies of interleukin-2- and interferon-gamma-producing T cells in patients with active Behcet’s disease. Invest. Ophthalmol. Vis. Sci..

[85] Alpsoy E., Cayirli C., Er H., Yilmaz E. (1998). The levels of plasma interleukin-2 and soluble interleukin-2R in Behcet’s disease: A marker of disease activity. J. Dermatol..

[86] Choi J.Y., Choi B., Shim J.A., Lee E.S., Kim D.Y., Bang D., Sohn S. (2015). IL-2/IL-2 antibody immune complex regulates HSV-induced inflammation through induction of IL-2 receptor alpha, beta, and gamma in a mouse model. Immunobiology.

[87] Castro I., Yu A., Dee M.J., Malek T.R. (2011). The basis of distinctive IL-2- and IL-15-dependent signaling: Weak CD122-dependent signaling favors CD8+ T central-memory cell survival but not t effector-memory cell development. J. Immunol..

[88] Choe J.Y., Lee H., Kim S.G., Kim M.J., Park S.H., Kim S.K. (2013). The distinct expressions of interleukin-15 and interleukin-15 receptor alpha in Behcet’s disease. Rheumatol. Int..

[89] Guo Y., Luan L., Rabacal W., Bohannon J.K., Fensterheim B.A., Hernandez A., Sherwood E.R. (2015). IL-15 superagonist-mediated immunotoxicity: Role of NK cells and IFN-γ. J. Immunol..

[90] Islam S.M.S., Choi B., Choi J., Lee E.-S., Sohn S. (2018). Frequencies of IL-15rα+ cells in patients with Behçet’s disease and the effects of overexpressing IL-15rα+ on disease symptoms in mice. Cytokine.

[91] Xu X.H., Shah P.K., Faure E., Equils O., Thomas L., Fishbein M.C., Luthringer D., Xu X.P., Rajavashisth T.B., Yano J. (2001). Toll-like receptor-4 is expressed by macrophages in murine and human lipid-rich atherosclerotic plaques and upregulated by oxidized LDL. Circulation.

[92] Nara K., Kurokawa M.S., Chiba S., Yoshikawa H., Tsukikawa S., Matsuda T., Suzuki N. (2008). Involvement of innate immunity in the pathogenesis of intestinal Behcet’s disease. Clin. Exp. Immunol..

[93] Do J.E., Kwon S.Y., Park S., Lee E.S. (2008). Effects of vitamin d on expression of Toll-like receptors of monocytes from patients with Behcet’s disease. Rheumatology.

[94] Pedersen L.B., Nashold F.E., Spach K.M., Hayes C.E. (2007). 1,25-dihydroxyvitamin D3 reverses experimental autoimmune encephalomyelitis by inhibiting chemokine synthesis and monocyte trafficking. J. Neurosci. Res..

[95] Sadeghi K., Wessner B., Laggner U., Ploder M., Tamandl D., Friedl J., Zugel U., Steinmeyer A., Pollak A., Roth E. (2006). Vitamin D3 down-regulates monocyte TLR expression and triggers hyporesponsiveness to pathogen-associated molecular patterns. Eur. J. Immunol..

[96] Choi B., Lee E.S., Sohn S. (2011). Vitamin D3 ameliorates herpes simplex virus-induced Behcet’s disease-like inflammation in a mouse model through down-regulation of Toll-like receptors. Clin. Exp. Rheumatol..

[97] Choi B., Suh C.H., Kim H.A., Sayeed H.M., Sohn S. (2017). The correlation of CD206, CD209, and disease severity in Behcet’s disease with arthritis. Med. Inflamm..

[98] Shannon B., Yi T.J., Thomas-Pavanel J., Chieza L., Janakiram P., Saunders M., Tharao W., Huibner S., Remis R., Rebbapragada A. (2014). Impact of asymptomatic herpes simplex virus type 2 infection on mucosal homing and immune cell subsets in the blood and female genital tract. J. Immunol..

[99] Choi B., Sayeed H.M., Islam S.M.S., Sohn S. (2017). Role of N-acetyl galactosamine-4-SO_4_, a ligand of CD206 in HSV-induced mouse model of Behcet’s disease. Eur. J. Pharmacol..

[100] Guasp P., Barnea E., Gonzalez-Escribano M.F., Jimenez-Reinoso A., Regueiro J.R., Admon A., Lopez de Castro J.A. (2017). The Behcet’s disease-associated variant of the aminopeptidase ERAP1 shapes a low-affinity HLA-B*51 peptidome by differential subpeptidome processing. J. Biol. Chem..

